# Detection of weak receptor–ligand interactions using IgM and J-chain-based fusion proteins

**DOI:** 10.1002/eji.201142151

**Published:** 2012-04-26

**Authors:** Johannes U Ammann, Martin Jahnke, Michael R Dyson, Jim Kaufman, John Trowsdale

**Affiliations:** 1 Department of Pathology, University of CambridgeCambridge, United Kingdom; 2 Department of Biochemistry, University of CambridgeCambridge, United Kingdom

**Keywords:** IgM, Immunoglobulin fusion protein, J-chain, Receptor-ligand

Dimeric IgG1 Fc tail-based fusion proteins are popular reagents in immunology. They are used for receptor activation, by mimicking ligands, and for meas-uring binding of proteins to receptors on cells. Using IgG fusion proteins, only those interactions with dissociation constants in the μM range or lower appear to be detected. Multimeric molecules, such as trimers, tetramers, and pentamers, have increased avidity but can be difficult to prepare and may have limited flexibility [1–2]. Multimerization of IgG fusion proteins using fluorescent beads can further increase avidity but in some applications, involving centrifugation for example, beads had in our hands a tendency to dissociate from cells in case of very weak interactions (unpublished observations).

In searching for a highly multimeric and flexible reagent for flow cytometry and immunoassays, we developed IgM-based fusion proteins [3–4]. IgM molecules may form “crab-like” or “table-like” structures when they bind antigen [5]. The antigen-binding domains act as “legs” and the rest of the molecule acts as a flexible structure connecting these antigen-bound sections. The adaptability of this structure may be due to the length of the heavy chain, which spans five immunoglobulin domains, whereas IgG encompasses four [6]. In principle each IgM multimer can have up to twelve antigen-binding sites whereas IgG molecules have only two. A further advantage of IgM over IgG molecules is that they are suitable for probing cells expressing IgG Fc receptors such as professional antigen presenting cells or NK cells.

We coupled the extracellular domains of proteins to Ig domains 3–5 of the human IgM heavy chain. Domains 4 and 5 of the human IgM heavy chain contain cysteine residues that form disulfide bonds, required for the formation of multimers [7–8]. The immunoglobulin IgG1 Fc tail monomer has two cysteine residues whereas the immunoglobulin IgM heavy chain has three. A 15 kDa molecule called the J-chain (joining-chain) is associated with IgA or IgM molecules. It contains eight cysteine residues that form disulfide bonds with cysteine residues in the IgM heavy chain [9]. Despite the fact that IgM molecules are secreted without the J-chain [10], it is important for formation of higher multimerized IgM molecules [11], and is required for trans-mucosal transport [12]. The number of J-chain molecules increases with the number of IgM heavy chains contained in a multimer [13]. However, the number of J-chain molecules per IgM polymer and the stoichiometry of the complexes is debated.

We assessed by flow cytometry and immunoassay whether co-expression of the J-chain influences binding of IgM-based heavy chain fusions to antigen. We also compared the binding of IgG-based and IgM-based fusion proteins by immunoassay.

We used a human IgM DNA sequence encoding the heavy chain domains 3–5, which Fukuda's laboratory used to detect binding partners of L-selectin and E-selectin by fluorescence microscopy [4]. IgG1a fusion proteins were encoded by SigpIg^+^ vectors (R&D Systems). The complementary deoxyribonucleic acid (cDNA) for mouse PD-L1 was from OpenBiosystems (Huntsville, AL, USA). The J-chain DNA sequence was from cDNA from the human B-cell line Raji, cloned into pCIPac DNA plasmid [14]. The DNA vector encoding the PD-1-rCD4 fusion protein was as published [15]. Fusion proteins (see Supporting Information Materials and Methods and Supporting Information Fig. 1 for detailed information) were expressed by transiently or stably transfected HEK-293 cells. When the J-chain was transiently co-transfected, a ratio of IgM fusion protein DNA to J-chain DNA of 10:1 was chosen. The concentration of the IgG or IgM fusion proteins in cell culture medium was measured using a standard sandwich enzyme-linked immunosorbent assay (ELISA) protocol. IgG or IgM fusions were found to be expressed at similar concentrations and were functional after storage for 1 year in Dulbecco's modified Eagle's Medium (DMEM) with 10% FCS and 0.01% azide at 4°C.

To assess the advantage of IgM + J-chain constructs by flow cytometry, we monitored binding of mouse PD-L1 molecule to CD3^+^ primary mouse T cells which express its ligand PD-1 when activated. PD-L1 binds PD-1 with a dissociation constant of 0.77 μM, and B7–1 of 1.4 μM [16]. We also used mouse BTN2A2, which binds to T cells through an unknown receptor [17]. First, we compared binding of PD-L1-IgG and BTN2A2-IgG fusion proteins with their IgM counterparts. PD-L1-IgG fusion proteins did not bind to nonactivated, CD3^+^ primary T cells and bound weakly to CD3^+^ primary T cells which had been activated for 3 days using 1 μg/ml anti-CD3 and 0.5 μg/ml anti-CD28 (Fig. 1A). PD-L1-IgM fusion proteins expressed without J-chain showed similar levels of binding. When binding of PD-L1-IgM fusion proteins was compared with binding of the same proteins co-expressed with the J-chain, the mean fluorescence intensity (MFI) of activated CD3^+^ T cells increased (Fig. 1A). As shown in Fig. 1B, no binding of BTN2A2-IgG or BTN2A2-IgM fusion proteins was detected to enriched CD3^+^ primary T cells even after activation. When the J-chain was co-expressed with the BTN2A2-IgM fusion protein, significant binding to the surface of activated CD3^+^ T cells was detected. Since the expression of the J-chain increased binding of IgM-based fusion proteins, we developed HEK-293 cell lines that were stably double-transfected with the respective IgM fusion plasmid, and the J-chain plasmid.

**Figure 1:**
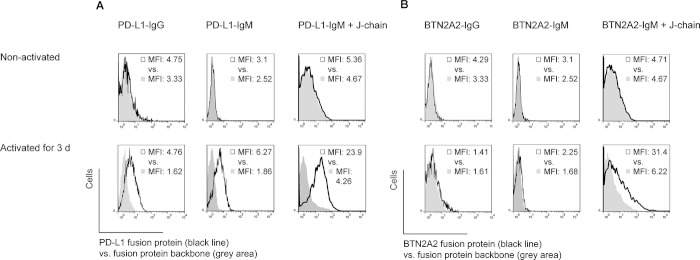
Binding of IgG and IgM fusion proteins containing the extracellular domains of mouse PD-L1 or BTN2A2 to enriched, CD3^+^ primary mouse T cells by flow cytometry. Where indicated, T cells were activated for 3 days in T75 flasks coated overnight with 1 μg/ml anti-CD3 and 0.5 μg/ml anti-CD28. The IgM or IgG part of the fusion protein alone was used as negative control (gray area); data from fusion protein is shown as the black line. After incubation for 1 h, bound fusion protein was detected by a biotinylated IgG- or IgM-specific antibody. Bound biotinylated antibodies were detected using fluorescently labeled streptavidin. Cells shown were gated on lymphocytes, single cells, and live cells.

To assess further the influence of the J-chain, a PerkinElmer DELFIA™ time-resolved fluorescence immunoassay system was used. First, the contribution of the J-chain to the binding of PD-L1-IgM fusion protein to plate-bound PD-1 was measured by immunoassay (Fig. 2A). About 2.5 times more PD-L1/PD-1 complexes were detected when the J-chain was co-expressed.

**Figure 2:**
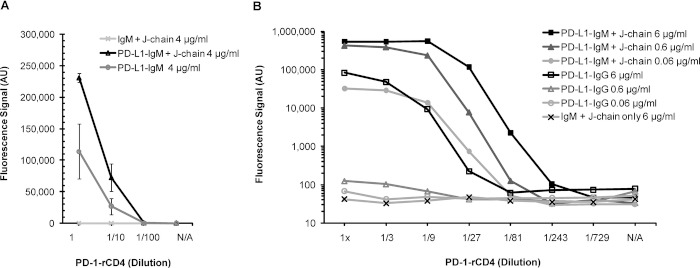
Binding of fusion proteins to plate-bound PD-1 by immunoassay. Bound IgG or IgM fusion proteins were detected using the same biotinylated antibodies as used for flow cytometry (Fig. 1) and bound biotinylated antibody was detected by streptavidin-Eu^3+^. Fluorescence of Eu^3+^-chelate complexes was measured using a time-resolved fluorescence protocol on a PerkinElmer Fusion plate reader. (A) Mean binding of the PD-L1-IgM fusion protein expressed with J-chain (black line) or without J-chain (dark gray line) in HEK-293 cells. Negative control: IgM backbone + J-chain (light gray line). PD-L1-IgM fusion proteins were incubated with PD-1-rCD4 for 1 h. Standard deviations were calculated from three samples. (B) Comparison of binding of IgM- with IgG-based fusions. Supernatants harvested from stably transfected HEK-293 cells in tenfold dilutions were incubated with plate-bound PD-1 for 2 h.

In addition, the binding to plate-bound PD-1 of dilutions of cell culture supernatant containing either PD-L1-IgG or PD-L1-IgM + J-chain was compared by immunoassay. When the PD-L1-IgM + J-chain complexes were used at 6 μg/ml, signals for bound PD-L1-IgM fusion protein exceeded, at high PD-1-rCD4 concentrations, the maximal detection limit of the immunoassay system, visible in Fig. 2B as a plateau. Neat PD-L1-IgG at 6 μg/ml showed a binding curve which was similar to that of PD-L1-IgM + J-chain at 0.06 μg/ml. This indicates that IgM + J-chain facilitate the binding of PD-L1 to PD-1 by a factor of approximately 100 as compared with the IgG backbone. Interestingly, similar amounts of undiluted PD-L1-IgG and PD-L1-IgM without J-chain bound to plate-bound PD-1, which agrees with the flow cytometry data (Fig. 1A versus Fig. 2).

When comparing the binding of supernatants with either 6 μg/ml PD-L1-IgG or PD-L1-IgM fusion protein and J-chain, a shift of the binding curve toward lower concentrations of PD-1 was observed (Fig. 2B). To quantify the observed shift, the dilution of PD-1-rCD4 at which a specific signal was detected was identified. A significant signal value was defined as threefold the value for unspecific binding. PD-L1-IgG detected PD-1 at a dilution of approximately 1 in 27, whereas for the PD-L1-IgM + J-chain, a dilution of approximately 1/243 was sufficient. This approximately tenfold difference enables detection of lower concentrations of the binding partner PD-1, possibly due to the higher flexibility and valency of the IgM backbone promoting binding and detection. Overall, the IgM + J chain increased binding of PD-L1 to PD-1 by approximately 1000-fold as observed at a 1/27 dilution of PD-1-rCD4 as compared with IgG.

In principle, IgM-based fusion proteins have advantages over IgG-, as they are more flexible and form higher multimers, promoted by the J-chain. We showed that co-expression of the J-chain increased binding of PD-L1-hIgM to PD-1 by approximately 2.5-fold. Compared with IgG, IgM + J-chain enabled detection of approximately tenfold lower amounts of binding partner. When we compared binding of different concentrations of fusion protein, we found that the IgM backbone strongly increased binding to plate-bound PD-1 by approximately 100-fold compared with IgG. A possible mechanism that could facilitate binding or reduce dissociation might be the higher valency of the IgM-backbone. In conclusion, IgM-based fusion proteins co-expressed with the J-chain appear to be useful reagents to characterize weak receptor–ligand interactions. In addition, our data indicate a function of the J-chain in facilitating IgM binding. It remains to be elucidated how coupling the IgM backbone and the J-chain facilitates formation of receptor–ligand interactions.

## Supplementary material

Disclaimer: Supplementary materials have been peer-reviewed but not copyedited.
