# Supplementary material for: Detection of weak receptor–ligand interactions using IgM and J-chain-based fusion proteins
Source: Eur J Immunol. 2012 Apr 26;42(5):1354–6. doi: 10.1002/eji.201142151 (PMC3437510; doi:10.1002/eji.201142151)

Supporting information

Materials and methods

IgM-based fusion proteins were generated by molecular cloning of the IgM backbone DNA sequence into the BTN2A2-IgG or PD-L1-IgG-encoding SigpIg+ vectors upstream of the IgG1 Fc part of the fusion protein. The secretory tailpiece of the human IgM heavy chain was also encoded in the human IgM heavy chain DNA sequence inserted (Mitoma J. et al. *Nat Immunol* .2007.**8**: 409-418). Expression of the IgG1 Fc tail was suppressed by including a stop codon at the end of the DNA sequence encoding the IgM domains. Primer sequences used for cloning the IgM heavy-chain were: forward- GTGTGGCGGCCGCTCCTGTGATTGCTGAGCTGC; backward-ATGGATCCTCAGTAGCAGGTGCCAGCTGT. The SigpIg+ vector containing the human IgM heavy chain domains 3-5 is illustrated in Supporting Information Figure 1. The J-chain DNA sequence was obtained from cDNA from the human B cell line Raji and cloned into the pCIPac DNA plasmid (Tregaskes C.A. et al. *Dev Comp Immunol* .2005.**29**: 361-374). Primer sequences used for cloning the J-chain were: forward-ATGAAGAACCATTTGCTTTTCTG; backward-TTAGTCAGGATAGCAGGCATCT.

IgM or IgG fusion proteins and J-chain were stably or transiently expressed in HEK-293 or HEK-293T cells as indicated. Fusion protein concentrations were measured using a standard ELISA protocol.

Flow cytometry analysis of binding of IgG fusion proteins or IgM fusion proteins containing the extracellular domains of PD-L1 or BTN2A2 to enriched, $CD3^+$ primary mouse T cells was performed as follows: Primary mouse $CD3^+$ T cells were enriched from spleen and lymph nodes of C57BL/6 mice by negative selection using a kit from Stem Cell Technologies (London, UK). Enriched cells were more than 95% positive for CD3. Where indicated, T cells were activated for 3 days in T75 flasks coated overnight with 1 μg/ml anti-CD3 clone 2C11 (eBioscience, Hatfield,

UK) and 0.5 µg/ml anti-CD28 clone 37.51 (eBioscience), at a cell density of 0.5x10^6 cells per ml in RPMI (Sigma-Aldrich, Gillingham, UK) with 10% FCS (PAA, Pashing, Austria), and 20 U/ml recombinant mouse IL-2 (Sigma-Aldrich) at 37°C and 5% CO_2. For staining of fusion protein, cells were incubated for 1 h with cell culture supernatant containing 2 µg/ml fusion protein at 37°C. The IgM or IgG part of the fusion protein alone was used as negative control. Bound fusion protein was detected by a biotinylated IgG or IgM-specific antibody (both Jackson Immunoresearch, West Grove/PA, USA) in 1% FCS PBS. Bound biotinylated antibody was detected using fluorescently labelled streptavidin (Sigma-Aldrich) in 1% FCS PBS. Bound fluorescent streptavidin was detected using a Cyan ADP flow cytometer (Beckman Coulter, High Wycombe, UK).

Immunoassay-based analysis of binding of PD-L1-IgG or IgM fusion proteins to plate-bound PD-1-CD4 was assessed using a Perkin Elmer (Cambridge, UK) FUSION plate reader. PD-1-CD4 fusion protein was immobilised to Nunc Maxisorp 96-well plates (ThermoFisher Scientific, Loughborough, UK) using an anti-rat CD4 antibody coated at 5 µg/ml (clone OX-68, AbD Serotec, Kidlington, UK) in PBS. Supernatants containing IgM or IgG fusion proteins were incubated with plate-bound PD-1-CD4 fusion proteins at 22°C for 1-2h as indicated. Bound IgG or IgM fusion proteins were detected using the same biotinylated antibodies as used for flow cytometry in PBS with 3% BSA and 0.1% Tween20. Bound biotinylated antibody was detected by DELFIA streptavidin-Eu^{3+} (Perkin Elmer) in PBS with 3% BSA and 0.1% Tween20. Fluorescence of Eu^{3+}-chelate complexes in DELFIA enhancement solution (Perkin Elmer) was measured using a DELFIA time-resolved fluorescence protocol on a Fusion plate reader.

Supporting Information Figure S1: The IgM-SigpIg+ fusion protein was generated by molecular cloning of the IgM backbone DNA sequence into SigpIg+ vector upstream of the IgG1

Fc part. Expression of the IgG1 Fc tail was suppressed by including a stop codon at the end of the DNA sequence encoding the IgM domains.

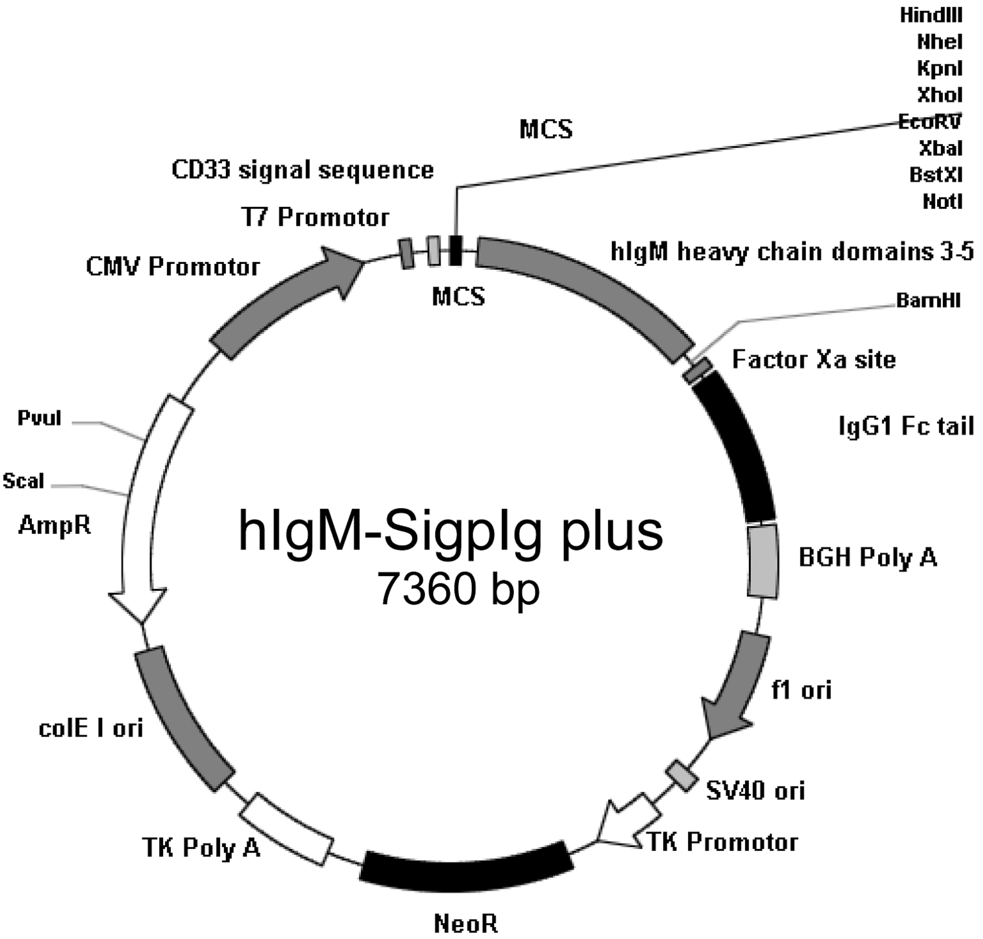

Supplement: Supplementary file 1 [file eji0042-1354-SD1.pdf]
